# Compulsive methamphetamine taking in the presence of punishment is associated with increased oxytocin expression in the nucleus accumbens of rats

**DOI:** 10.1038/s41598-017-08898-8

**Published:** 2017-08-21

**Authors:** Irina N. Krasnova, Maria Carla Gerra, Donna Walther, Subramaniam Jayanthi, Bruce Ladenheim, Michael T. McCoy, Christie Brannock, Jean Lud Cadet

**Affiliations:** Molecular Neuropsychiatry Research Branch, DHHS/NIH/NIDA Intramural Research Program, 251 Bayview Boulevard, Baltimore, MD 21224 USA

## Abstract

Methamphetamine addiction is mimicked in rats that self-administer the drug. However, these self-administration (SA) models do not include adverse consequences that are necessary to reach a diagnosis of addiction in humans. Herein, we measured genome-wide transcriptional consequences of methamphetamine SA and footshocks in the rat brain. We trained rats to self-administer methamphetamine for 20 days. Thereafter, lever-presses for methamphetamine were punished by mild footshocks for 5 days. Response-contingent punishment significantly reduced methamphetamine taking in some rats (shock-sensitive, SS) but not in others (shock-resistant, SR). Rats also underwent extinction test at one day and 30 days after the last shock session. Rats were euthanized one day after the second extinction test and the nucleus accumbens (NAc) and dorsal striatum were collected to measure gene expression with microarray analysis. In the NAc, there were changes in the expression of 13 genes in the SRvsControl and 9 genes in the SRvsSS comparison. In the striatum, there were 9 (6 up, 3 down) affected genes in the SRvsSS comparison. Among the upregulated genes was oxytocin in the NAc and CARTpt in the striatum of SR rats. These observations support a regional role of neuropeptides in the brain after a long withdrawal interval when animals show incubation of methamphetamine craving.

## Introduction

Methamphetamine (METH) addiction is a biopsychosocial disorder with a very high prevalence throughout the world^1^. Its abuse is associated with negative impact on the brain and peripheral organs^2, 3^. These adverse consequences include abnormal blood flow and brain structural pathologies^4–6^, with consequent neuropsychological deficits^7^. Although a diversity of drugs can cause the neuropsychiatric syndromes designated as addictions, it is important to identify specific neurobiological processes involved in causing compulsive taking of individual substances. This is relevant to the development of pharmacological agents against addictions because individual substances may impact the brain differentially^4^. This suggests the importance of identifying and detailing the potential impact of these drugs on distributed systems both within and beyond the so-called reward circuitry^8^ because changes in these systems might influence or dictate treatment approaches^9–11^. These statements suggest that addiction may occur as the result of repeated exposure to drugs that lead to neuropathological changes in distributed networks of potentially dissociable reward and non-reward pathways in the mammalian brain^4, 8^. This conclusion is supported by accumulated evidence from epigenetic, transcriptional, neuroimaging, and neuropsychological studies that have indeed provided support for a role of various brain regions in the development and maintenance of addicted states^7, 10, 12–14^. This suggestion also envisions the development of preclinical models that include additional criteria because, by definition, addiction diagnoses include more than just drug SA. Improved models may allow for the identification of diverse neuroanatomical structures, epigenetic and transcriptional events, and memory processes that might be involved in the promotion of addiction to specific substances such as methamphetamine.

A better understanding of how individual drugs differentially impact specific brain regions within these distributed networks will require more in-depth knowledge of the specific biochemical and molecular consequences of each addictive drug on different brain regions^4^. This statement thus hints to the need to decipher molecular changes that may be predictive of resilience to and/or of risk to develop an addictive state^9^. Elucidation of these molecular signatures has the potential to help in the development of pharmacological interventions to modify the clinical manifestations of substance use disorders. To address these issues, we have begun to use a model that uses footshocks to differentiate rats that continue to self-administer methamphetamine compulsively from those that significantly reduce their intake in the presence of punishment^15–17^. In the present study, we have used this model to investigate transcriptional changes in the nucleus accumbens (NAc) and dorsal striatum after a month of forced abstinence.

## Results

### Behaviors

**Figure 1:**
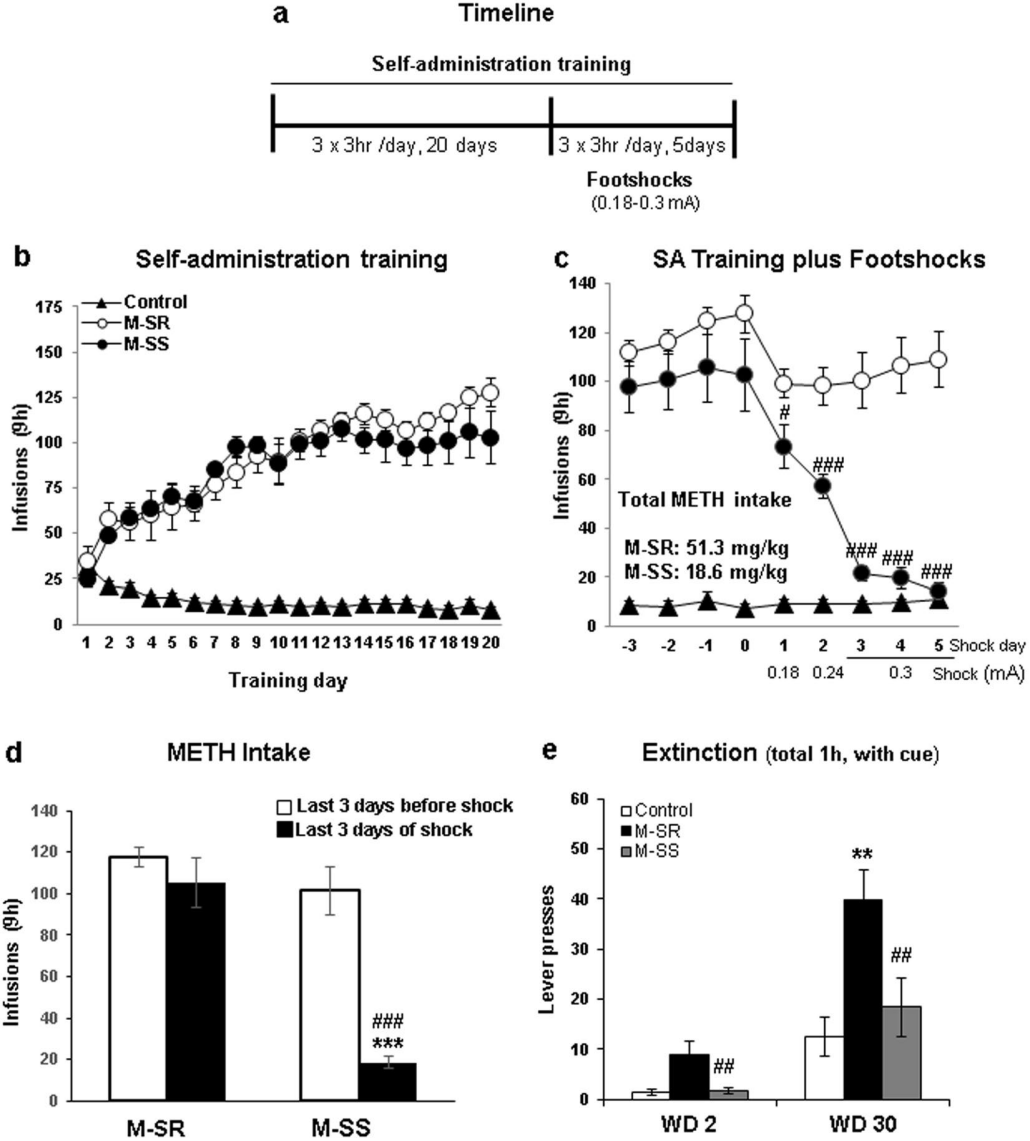
Extended access to methamphetamine and footshock punishment lead to compulsive methamphetamine taking or abstinence in rats. (**a**) Timeline of behavioral experiments. (**b**) Rats with long access to METH escalate drug self-administration. The groups were separated post-facto based on their responses to footshocks. (**c**) Increasing footshock intensity leads to reduction of METH intake in SS but not in SR animals. (**d**) Mean drug intake on the last 3 days of METH SA compared to last 3 days of shocks. (**e**) Rats show incubation of METH craving at withdrawal day (WD) 30 in comparison to WD2. SR rats showed higher lever pressing at WD30 in comparison to the SS rats.

Figure 1a shows the timeline of the behavioral experiments. METH-trained rats increased their drug intake and control rats decreased lever pressing for saline during the SA training phase (Fig. 1b). The repeated measures ANOVA for earned infusions included the between-subject factor reward type (saline, METH) and the within-subject factor of SA day (training days 1–20). The analysis showed a significant effect of training day x reward type [F(19,456) = 25.31, p < 0.0001]. The significant interaction reflects the fact that METH-trained rats continued to increase their drug intake over the training days whereas the saline rats decreased and stabilized their intake to very low values (Fig. 1b).

During the training plus punishment phase, footshock intensity was increased from 0.18 to 0.30 A over a period of 5 days (Fig. 1c). The increased shock intensity caused reinforced responding to significantly decrease in the shock-sensitive (SS) but not in the shock-resistant (SR) rats (Fig. 1c). The statistical analysis of METH infusions earned included the between-subject factor of groups [(SR) and (SS)] and the within-subject factor of shockday (shockdays 1–5). There was a significant effect of shockday x group [F(4,56) = 16.95, p < 0.0001], with the highest intensity (0.30 mA) showing almost total suppression of METH intake in the SS rats (Fig. 1c). Figure 1d shows the effects of the highest intensity (0.30 mA, 3 days of footshocks) on METH intake in comparison to the last 3 days of training before application of foot-shocks. Rats that were yoked to SR rats to receive non-contingent footshocks (YSR) received significant more footshocks than rats yoked (YSS) to the SS group [(291 ± 39 vs 94 ± 13, respectively), p < 0.001].

We also measured cue-induced reinstatement at withdrawal days 2 and 30 (Fig. 1e) and found that both SR and SS animals increased lever pressing at WD 30 in comparison to WD2. In addition, the SS rats showed less lever pressing than the SR rats at both time points.

### Microarray Analyses

We used the Affymetrix array platform that contain a total of 68,842 probes to measure transcriptional changes in the NAc and dorsal striatum of animals euthanized one day after the second extinction test. These probes consist of 24,753 protein coding, 28,724 noncoding, and unassigned pseudogenes. The analysis of raw array data revealed that 9044 genes were expressed in the NAc and 9754 genes were expressed in the dorsal striatum. This was determined by comparing the average signal values of transcripts in the control animals to the average signal value of the bioB gene in the control animals. Gene with average signals above the average signal value of the bioB gene were considered as expressed in the specific brain region. The expressed genes were included in further analyses of differential gene expression between the experimental groups. There were 181 genes differentially expressed in the NAc and 304 genes were differentially expressed in the dorsal striatum.

**Figure 2:**
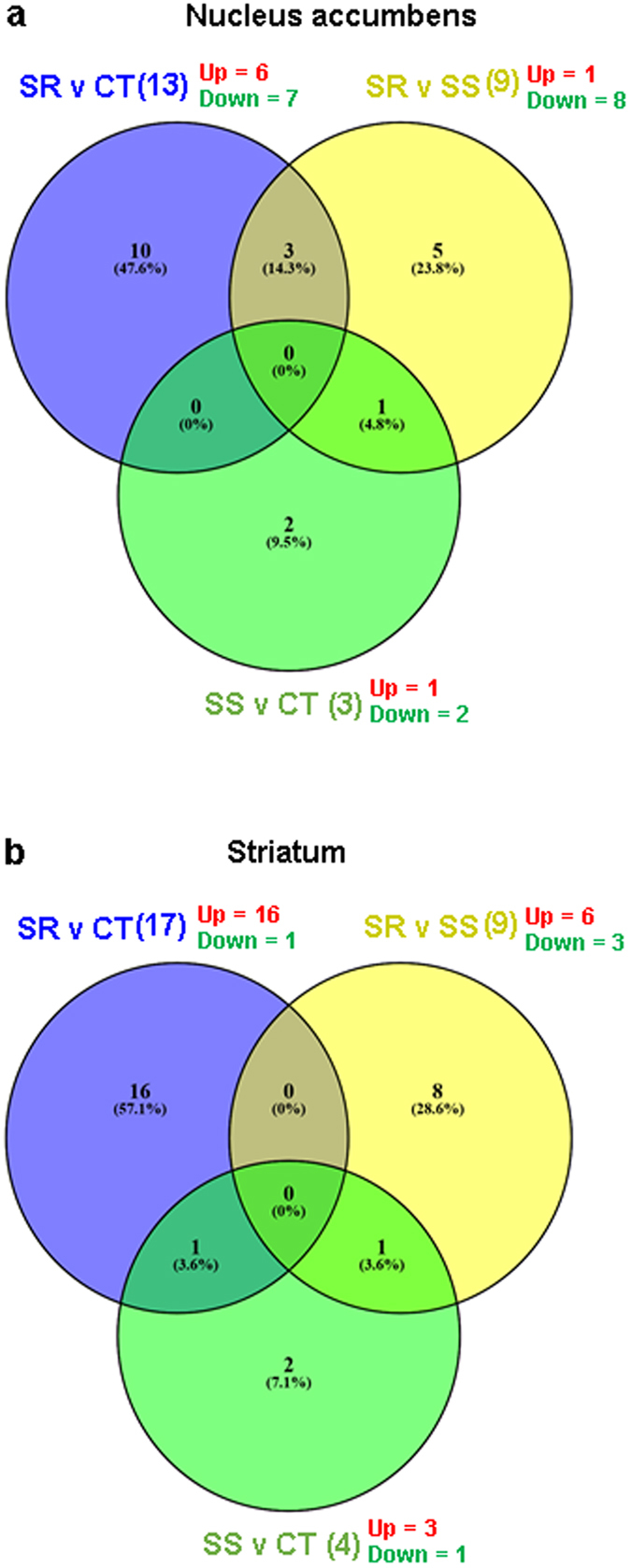
Microarray analysis of gene expression in the NAc and dorsal striatum of rats after one month of withdrawal from methamphetamine SA. Comparison of gene expression in pairwise comparisons between SR, SS, and control rats in NAc (**a**) in dorsal striatum (**b**) [n = 6 in each group]. The number of up- and downregulated genes in each comparison is shown in red and green, respectively. The list of genes and their classification are shown in Tables 1 and 2.

**Figure 3:**
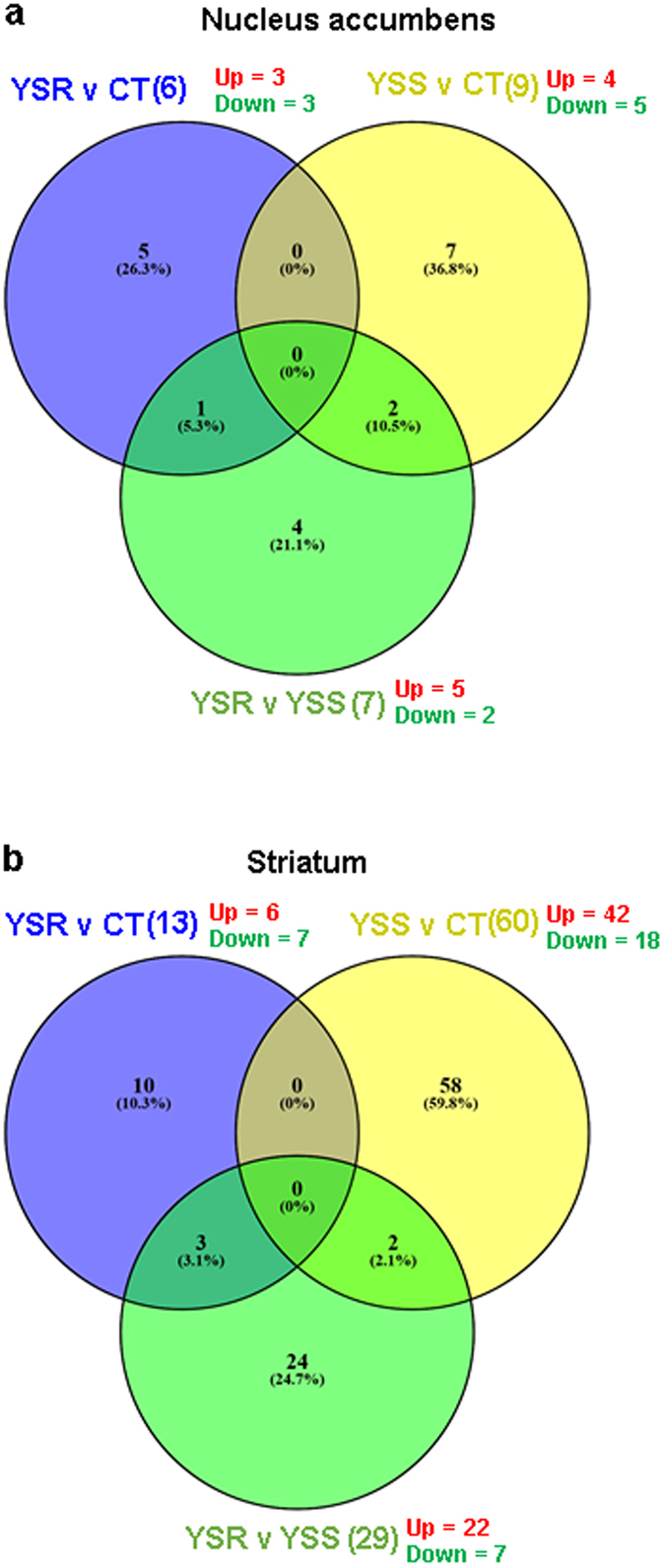
Microarray analysis of gene expression in the NAc and striatum of rats after one month of withdrawal from methamphetamine SA. Comparison of gene expression in pairwise comparisons between YSR (n = 3), YSS (n = 3), and control (n = 6) rats in NAc (**a**) in dorsal striatum (**b**). The number of up- and downregulated genes in each comparison is shown in red and green, respectively. The list of genes and their classification are shown in Tables 3 and 4.

The results of the microarray analyses in the NAc are shown in Figs 2a and 3a, Table 1, and in supplementary Tables S1 and S1. There were multiple pairwise comparisons that included: shock-resistant versus control (SRvsCT), shock-sensitive versus CT (SSvsCT), and SR versus SS (SRvsSS) groups, yoked SR versus CT (YSRvsCT), yoked SS versus CT (YSRvsCT), and YSRvsYSS (see Figs 2a and 3a and Table 1, and in Supplementary Tables S1 and S1). The differences in gene expression between YSR and YSS are shown in the Supplementary Table S2. In the NAc, there were 13 (6 up- and 7 downregulated) differentially expressed transcripts in the SRvsCT comparison (±1.7-fold change, p < 0.05) (Fig. 2a). The SSvsCT comparison contained only 3 (1 up and 2 down) differentially expressed genes while the SRvsSS comparison had 9 (1 up-and 8 downregulated) differentially expressed transcripts. We used DAVID and literature searches to generate functional annotation and classification analyses for significantly expressed transcripts. The names of these genes and their classification are shown in Table 1. The list includes genes that participate in metabolism and signal transduction as well as several microRNA precursor transcripts. Genes of interest in the SRvsCT comparison include oxytocin that showed 6.19-fold increases in the SR group. OXT was also increased in the SR in comparison to the SS group (5.71-fold) (Table 1). The yoked animals also showed some changes in gene expression in comparison to control (Fig. 3a). Rats yoked to the SR group showed changes in 6 transcripts (3 up and 3 up) in comparison to control. Animals yoked to the SS group showed 9 (4 up and 5 down) differentially expressed genes. The list of genes also included genes involved in metabolism, phosphorylation cascades, and signal transduction (Supplementary Table S1).

**Table 1:** Classification of NAc genes with altered expression after METH SA and footshocks.

GenBank Accession	Symbol	Description	Fold Change SR vs CT	Fold Change SS vs CT	Fold Change SR vs SS
***Metabolism***					
NM_001037355	Mettl7a	methyltransferase like 7 A	1.68	1.25	1.35
***MicroRNA***					
NR_030567	Mir466f-2	microRNA 466f-2	−4.71	−1.03	−4.55
MTA_TR0500009144.mm	Mir1191	microRNA 1191	−1.47	1.12	−1.65
MTA_TR0100005862.mm	Mir297c	microRNA 297c	−2.20	1.12	−2.46
MTA_TR0200016496.mm	Mir3098	microRNA 3098	1.02	2.78	−2.72
MTA_TR1300007696.mm	Mir325	microRNA 325	−1.75	1.01	−1.77
NR_030571	Mir467c	microRNA 467c	−1.71	1.23	−2.10
NR_030645	Mir467e	microRNA 467e	−1.66	1.19	−1.98
MTA_TR0200000271.mm	Mir669f	microRNA 669 f	−1.86	−1.13	−1.64
NR_032296	Mir770	microRNA 770	1.96	1.64	1.20
NR_032756	Mir876	microRNA 876	−1.85	−1.18	−1.57
NR_032286	Mir598	microRNA mir-598	−1.25	1.35	−1.69
MTA_TR0500000814.mm	Mir362	microRNA mir-6362	−2.38	−1.09	−2.19
NR_105852	Mir6481	microRNA mir-6481	−1.83	−1.47	−1.25
***Protein Kinase/Phosphatase***					
NM_031769	Pdxk	pyridoxal (pyridoxine, vitamin B6) kinase; similar to pyridoxal (pyridoxine, vitamin B6) kinase; pyridoxal kinase-like	1.73	1.63	1.07
***Proteolysis***					
NM_020091	Cym	chymosin	−1.38	−2.39	1.74
***Signal Transduction***					
NM_001191104	Tmem223	transmembrane protein 223	2.07	1.31	1.58
NM_022005	Fxyd6	FXYD domain-containing ion transport regulator 6	2.10	1.35	1.56
NM_053859	Slc17a7	solute carrier family 17 (vesicular glutamate transporter), member 7	−1.50	−3.06	2.04
NM_012996	Oxt	oxytocin/neurophysin 1 prepropeptide	6.19	1.08	5.71
***Synaptic Plasticity***					
MTA_TR1100003798.mm	Zfp616	zinc finger protein 616	−1.81	−1.56	−1.16

The results of the striatal microarray data are shown in Figs 2b and 3b, Table 2, and in supplementary Tables S3 and S4. Figure 2b shows that there were 17 (16 up and 1 down) differentially expressed transcripts in the SRvsCT comparison, 4 (3 up and 1 down) transcripts in the SSvsCT comparison, and 9 (6 up and 3 down) transcripts in the SRvsSS comparison. Table 2 shows a list of genes that participate cell adhesion, metabolism, signal transduction, and transcription regulation in the dorsal striatum. Figure 3b shows the results of the comparison between the yoked animals and the control group. There were 13 (6 up and 7 down) differentially expressed transcripts in the YSRvsCT comparison, 60 (42 up and 18 down) in the YSSvsCT comparison and 29 (22 up and 7 down) differentially expressed genes in the YSRvs YSS comparison. The genes are listed in supplementary Table S3. The differences in gene expression between YSR and YSS in the dorsal striatum are shown in Supplementary Table S4.

**Table 2:** Classification of striatal genes with differential expression after METH SA and footshocks.

Genbank Accession	Gene Symbol	Description	Fold Change SR vs CT	Fold Change SS vs CT	Fold Change SR vs SS
***Calcium Ion Binding***					
NM_001271195	Fstl5	follistatin-like 5	1.52	−1.11	1.68
***Cell Adhesion***					
NM_001169129	Pcdh19	protocadherin 19	2.11	1.50	1.41
XM_001061943.6	Cdh4	cadherin 4	2.02	1.43	1.41
***DNA Binding***					
NM_153626	Npas4	neuronal PAS domain protein 4	−1.75	−1.48	−1.18
***Membrane Structure***					
NM_001191104	Tmem22	transmembrane protein 223	1.98	1.30	1.52
NM_001109561	Nrsn2	neurensin 2	1.40	−1.24	1.74
***Metabolism***					
NM_001107311	Enpp6	ectonucleotide pyrophosphatase/phosphodiesterase 6	1.12	−1.78	1.99
NM_001107671	Plcxd3	phosphatidylinositol-specific phospholipase C, X domain	1.69	1.22	1.39
NM_001191118	Siah3	siah E3 ubiquitin protein ligase family member 3	1.75	1.25	1.41
NM_001258237	Ace3	angiotensin I converting enzyme (peptidyl-dipeptidase A	−1.14	1.54	−1.75
NM_031655	Lxn	latexin	1.05	1.71	−1.63
NM_031776	Gda	guanine deaminase	1.81	1.33	1.36
XM_006247643.3	LOC102556346	angiotensin-converting enzyme-like	−1.18	1.87	−2.21
***MicroRNA***					
NR_035479	Mir1954	microRNA 1954	−1.14	−1.88	1.66
NR_106093	Mir7234	microRNA mir-7234	−1.42	1.34	−1.91
***Protein Binding***					
NM_017110	Cartpt	CART prepropeptide	1.33	−1.59	2.11
***Signal Transduction***					
XM_008763452.2	Eps8	epidermal growth factor receptor pathway substrate 8	1.89	1.35	1.40
NM_001102381	Nts	neurotensin	1.82	1.13	1.61
NM_001108774	Mrap2	melanocortin 2 receptor accessory protein 2	1.73	1.44	1.20
NM_001109018	Rab9b	RAB9B, member RAS oncogene family	1.66	1.06	1.57
NM_001287022	Rgs21	regulator of G-protein signaling 21	1.01	1.89	−1.87
NM_012568	Glra2	glycine receptor, alpha 2	3.50	2.07	1.69
NM_017362	Chrm5	cholinergic receptor, muscarinic 5	3.06	1.58	1.93
NM_022669	Scg2	secretogranin II	1.81	1.25	1.45
NM_031823	Wfs1	Wolfram syndrome 1 (wolframin)	2.35	1.68	1.40
***Transcription***					
NM_001107473	Zim1	zinc finger, imprinted 1	1.78	1.14	1.57
NM_001191702	Zfhx4	zinc finger homeobox 4; Unannotated AceView Transcript;	1.73	1.24	1.39
XM_008762737.1	PEG10	ENCODES a protein that exhibits poly(A) RNA binding	1.71	1.02	1.67

### Validation of array-identified genes by quantitative PCR

#### Compulsive METH taking-related genes

**Figure 4:**
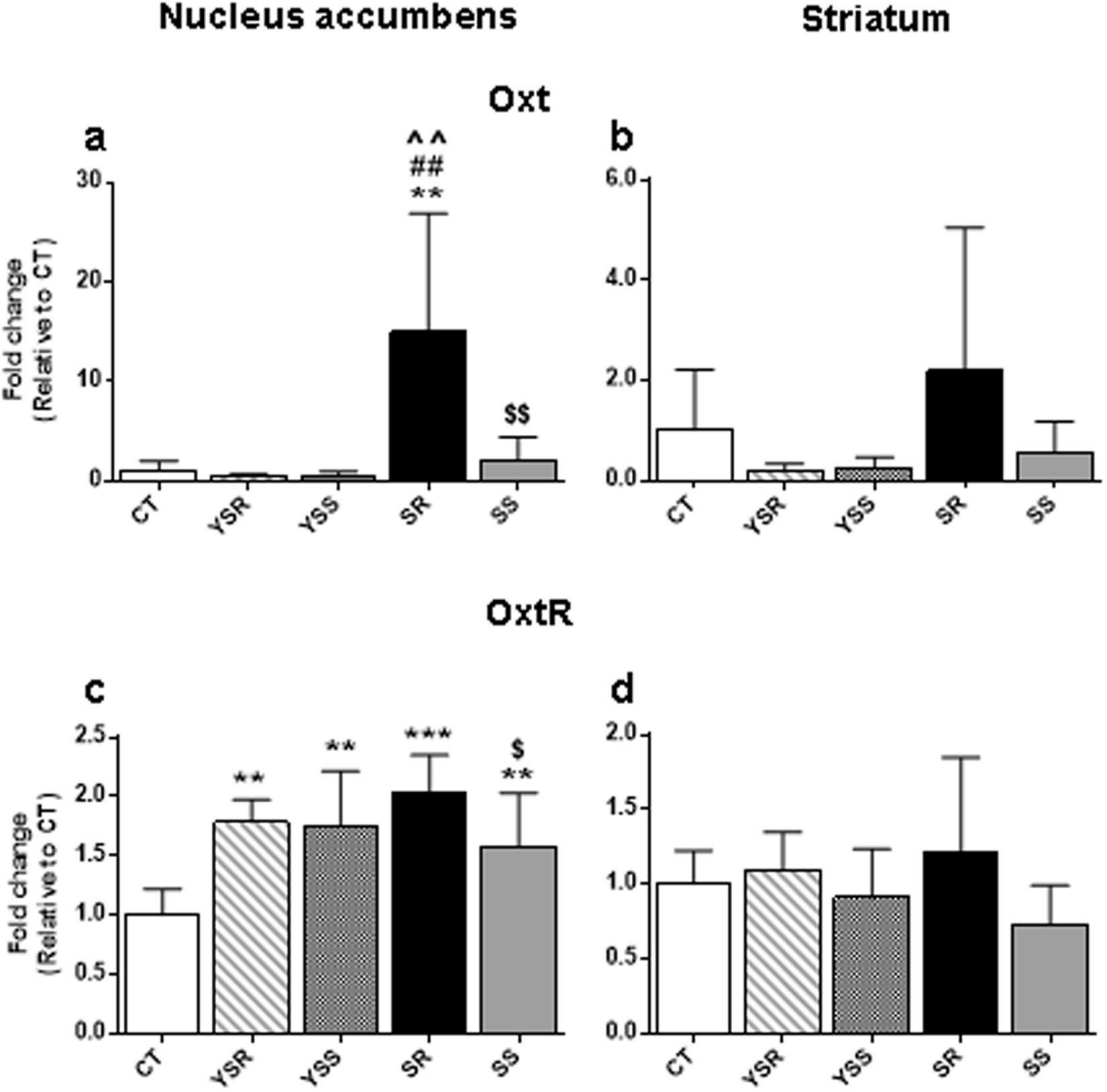
PCR validation of changes in oxytocin mRNA levels in the rat NAc after a month withdrawal from methamphetamine SA and footshocks. We conducted quantitative PCR using individual RNA from the NAc (**a**,**c**) and dorsal striatum (**b**,**d**) of rats from the various conditions (CT, n = 6–8; SR, n = 6–8; SS, n = 6–9; YSR, n = 3; YSS, n = 3–6). The shock-resistant rats showed significant increases in oxytocin (**a**) and oxytocin receptor (**c**) in the NAc but not in the dorsal striatum (**b** and **d**, respectively). Values are means ± SEM fold changes relative to the control group. Key to statistics: **p < 0.01, ***p < 0.001, in comparison to the control group; ##p < 0.01, in comparison to YSR rats; ^^p < 0.01, in comparison to the YSS group; ^$^p < 0.05, ^$$^p < 0.01 in comparison to the SR group.

To validate the changes in oxytocin observed in the microarray data, we ran quantitative PCR using RNAs from the various groups of rats. Figure 4 presents the effects of METH SA and footshocks on the expression of oxytocin (OXT) in the nucleus accumbens and dorsal striatum. NAc OXT mRNA levels displayed significant changes [F(4,19) = 5.44, p = 0.0043] in expression. Post-hoc analyses demonstrated that the SR group showed substantial increases in OXT mRNA expression in comparison to the CT, SS, and yoked animals (Fig. 4a). YSR and YSS groups showed small decreases that were not significant in comparison to the control group (Fig. 4a). OXT receptor (OXTR) mRNA levels also exhibited significant changes [F(4,19) = 7.19, p = 0.0011] after withdrawal from METH SA, with post-hoc analyses indicating that all groups showed small increases in comparison to the control group (Fig. 4b). In contrast, there were no significant changes in OXT [F(4,24) = 1.643, p = 0.1961] or OXTR [F(4,27) = 1.715, p = 0.1767] mRNA levels in the dorsal striatum (Fig. 4b and d, respectively).

**Figure 5:**
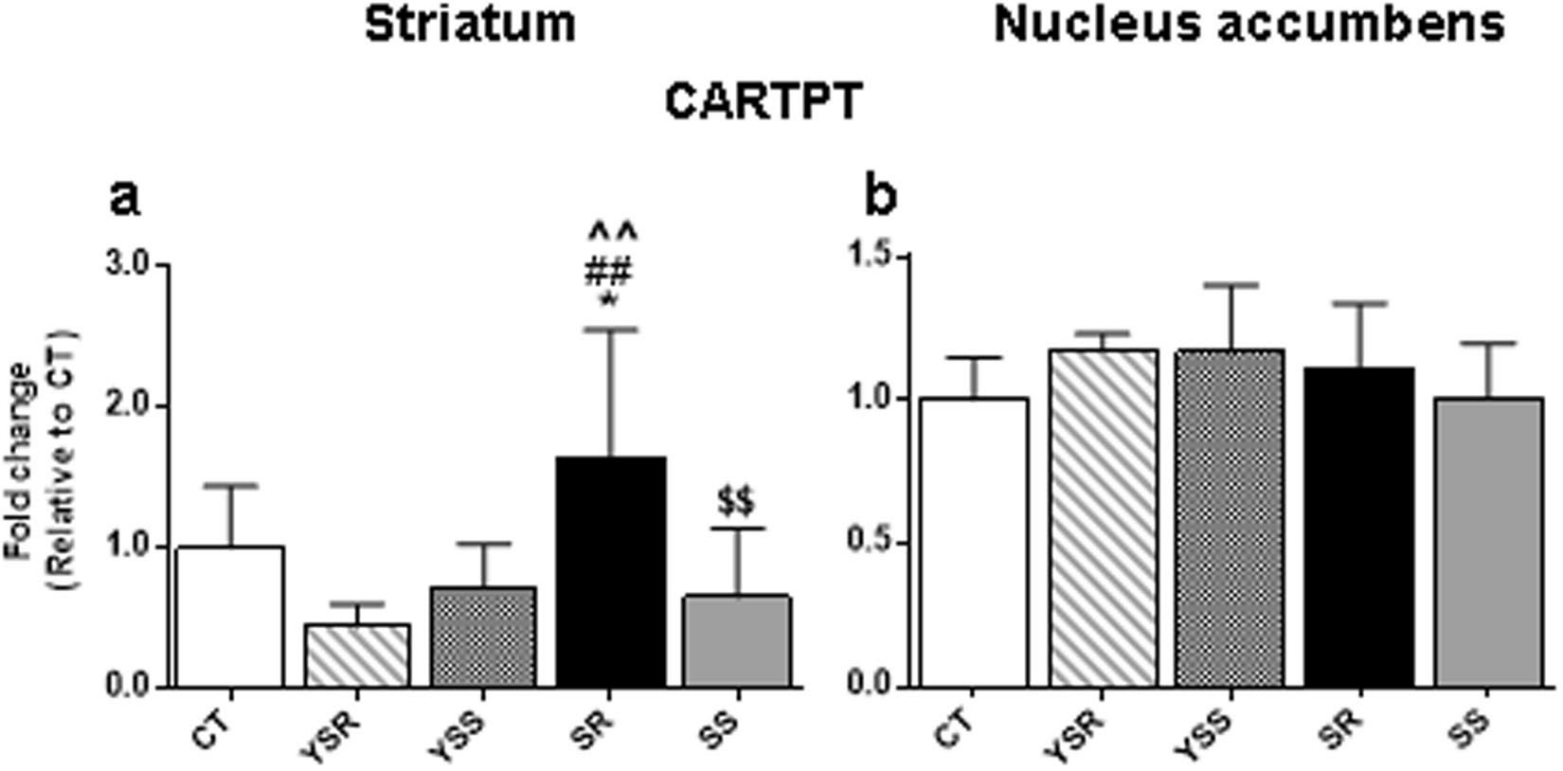
PCR validation of changes in CARTpt mRNA expression in the dorsal striatum after a month withdrawal from methamphetamine SA and footshocks. We conducted quantitative PCR using individual RNA from the dorsal striatum (**a**) and NAc (**b**) of rats. The shock-resistant rats showed significant increases in CARTpt in the dorsal striatum (**a**) but not in the NAc (**b**). Values are means ± SEM fold changes relative to the control group. Key to statistics: *p < 0.05, in comparison to the control group; ##p < 0.01, in comparison to YSR rats; ^^p < 0.01, in comparison to the YSS group; $$p < 0.01 in comparison to the SR group.

We also validated the expression of striatal CARTpt that showed increased expression between SR and SS in the microarray data (Table 2). Figure 5a showed confirmation of the significant [F(4,28) = 4.220, p = 0.0085] increases in CARTpt in the striatum of SR rats. There were, however, no significant changes in the NAc [F(4,26) = 1.059, p = 0.3966] (Fig. 5b).

#### Footshock-responsive genes

**Figure 6:**
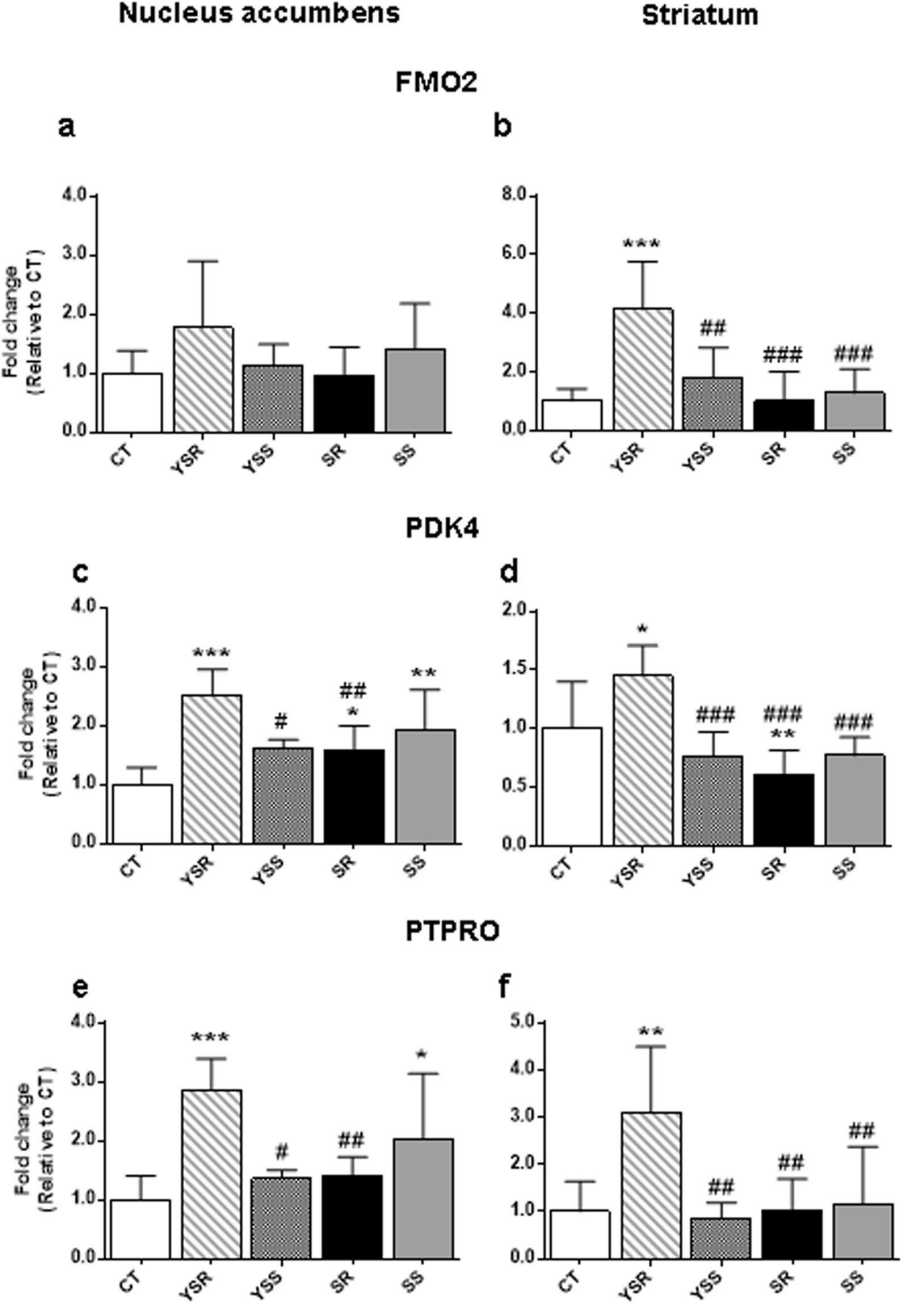
PCR measures of changes in FMO2, PDK4, and PTPRO mRNA levels in the rat NAc and dorsal striatum after a month withdrawal from methamphetamine SA and footshocks. We conducted quantitative PCR using individual RNA from the NAc (**a**,**c**,**e**) and dorsal striatum (**b**,**d**,**f**) of rats from the 5 groups of rats. The YSR that received many non-contingent footshocks (291 ± 39) showed significant increases in FMO2 in the dorsal striatum (**b**) but not in the NAc (**a**). YSR rats also experienced increases in PDK4 in both the NAc (**c**) and dorsal striatum (**d**). PTPRO mRNA levels were also increased in the NAc (**e**) and dorsal striatum (**f**) of YSR rats. Values are means ± SEM fold changes relative to the control group. Key to statistics: *p < 0.05, **p < 0.01, ***p < 0.001, in comparison to the control group; #p < 0.01, ##p < 0.01, ###p < 0.001, in comparison to the YSR rats.

We also validated the expression of some genes that showed changes in rats that were yoked to the METH SA to receive similar number of footshocks as the METH SA animals (Fig. 6). Figure 6a and b show that animals that received footshocks experienced no significant changes in FMO2 (flavin-containing monooxygenase) in the NAc but showed significant [F(4,19) = 6.849, p = 0.0014] changes in the dorsal striatum. Post-hoc analyses showed that the YSR group (291 ± 39 footshocks) exhibited significantly higher mRNA levels in the dorsal striatum than the other groups (Fig. 6b). Figure 6c and d show that there were significant changes in the expression of PDK4 (pyruvate dehydrogenase kinase 4) in the NAc [F(4,19) = 6.388, p = 0.0020] and dorsal striatum [F(4,28) = 6.497, p = 0.0008]. In addition to the YSR group showing higher levels than the other groups in the NAc, post-hoc analyses showed that both SR and SS groups [but not the YSS (94 ± 13 footshocks) rats] also showed small increases in comparison to the control animals. The results suggest that METH might have partially attenuated the transcriptional effects of the large number of footshocks that the SR rats had received (compare YSR to SR results in Fig. 6c). In the dorsal striatum (Fig. 6d), YSR rats also showed higher expression than the other groups whereas the SR animals showed lower expression than the control group, suggesting the large amount of METH taken by the SR rats might have significantly suppressed the effects of footshocks alone (YSR) in this brain region. Smaller number of footshocks and lower amount of METH (SS) did not significantly influence the expression of PDK4 in the dorsal striatum (Fig. 6d). These data support the notion that the NAc and dorsal striatum may respond differentially to a diversity of exogenous stimuli.

Figure 6e and f show the effects of METH and footshocks on the expression of PTPRO (protein tyrosine phosphatase, receptor type O) in the NAc and dorsal striatum, respectively. PTPRO mRNA expression was significantly [F(4.19) = 4.890, p = 0.0070] affected in the NAc. Post-hoc analyses showed that the YSR and SS groups exhibited higher mRNA expression than controls (Fig. 6e). In addition, SR rats showed lower expression than YSR animals, again suggesting that large amount of METH might have inhibited the effects of footshocks alone on PTPRO mRNA levels in the NAc. In the dorsal striatum, there were also significant [F(4,28) = 3.982, p = 0.0111] increases in the levels of PTPRO mRNA in the YSR group when compared to the other groups (Fig. 6f). Post-hoc tests indicated that PTPRO mRNA levels in the other groups were comparable to those of controls (Fig. 6f).

## Discussion

The aim of the present study was to use an unbiased genome-wide approach to identify transcriptional changes in shock-sensitive and -resistant rats that had all undergone METH self-administration training and footshocks. We also compared gene expression in rats that were yoked to the METH SA animals to control for the effects of footshocks during the shock phase of the study. Our aim was to compare gene expression in the NAc and dorsal striatum because these two brain regions are known for their involvement in different aspects and phases of compulsive drug-taking behaviors and during withdrawal from METH SA^16, 18–24^. We had reasoned that drug-induced alterations of these neuroanatomical systems may negatively impact information processing and, over time, might have led to disturbed responses to environmental cues^7^, based on their different connections to various brain regions implicated in addiction^25^. Indeed, we found significant increases in oxytocin mRNA levels in the NAc, but not in the dorsal striatum, 30 days after stopping METH SA. These results are consistent with those of Baracz *et al*.^26^ who had reported significant increases in plasma oxytocin levels immediately after and at 15 days of withdrawal from METH self-administration. The observation that animals with reduced METH intake in the presence of footshocks did not show significant increases in oxytocin mRNA levels suggest that these observations may be related specifically to compulsive METH takers that continue to take the drug despite the footshocks (SR rats). The changes in oxytocin mRNA are also not secondary to footshocks alone since the yoked animals did not show any increases in oxytocin in comparison to control rats. We also found significant increases in CARTpt mRNA levels in the dorsal striatum but not in the NAc of compulsive METH takers, suggesting that different neurotransmitter systems may be activated in different brain regions during protracted forced abstinence from METH self-administration.

### Potential role of NAc oxytocin in compulsive methamphetamine taking

Oxytocin is a centrally active neuropeptide that is involved in social bonding/attachment^27, 28^ and in some psychopathological states including stress, affective disorders, and schizophrenia^29, 30^. A potential role for oxytocin in addiction has also been investigated by several groups of scientists^18, 26, 31–34^. For example, intracerebroventricular injections of oxytocin were shown to reduce METH-induced increased locomotor activity^35^ and conditioned place preference (CPP)^36^ in mice. Carson *et al*.^32^ have also reported that peripheral oxytocin administration can dose-dependently reduce the number of self-administered METH infusions by rats. Oxytocin also reduced locomotor activity and drug-induced reinstatement^32^. Follow-up studies have also documented that infusions of oxytocin in the NAc can reduce METH CPP^37^ and METH-primed reinstatement^31^. These studies are consistent with the recent report that oxytocin, given peripherally or intracerebrally, can reduce METH demand and seeking by rats^33^. When taken together, these observations implicate an important role of this neuropeptide in the effects of this psychostimulant. This conclusion is consistent with the report that METH can cause increased plasma oxytocin levels and decreased oxytocin receptors in the NAc of rodents^31^. Thus, our findings of increased oxytocin mRNA levels in the NAc may constitute compensatory responses to replace oxytocin that might have been released from the NAc during compulsive METH taking.

### Potential role of striatal CARTpt in withdrawal from compulsive methamphetamine taking

We also found significant increases in CARTpt mRNA levels in the striatum, but not in the NAc, after 30 days of withdrawal from METH (see Fig. 5). These results are consistent with recent proposals that the dorsal striatum may be involved in longterm abstinence from METH SA^20, 38^. CARTpt is a brain/gut neuropeptide initially identified by differential display in the dorsal striatum of rats that were injected with either cocaine or amphetamine^39^. In the NAc, CARTpt was reported to be localized in D1-containing neurons not in D2 neurons^40^. In addition to the NAc, CARTpt is widely distributed in the brain^41, 42^ and appears to play a role in the acute and chronic effects of some drugs of abuse^43, 44^. For example, intra-NAc administration of CART was shown to attenuate hyperactivity induced by either cocaine or amphetamine^45, 46^. The effects of CARTpt were reported to be dependent on the modulation of both dopamine (DA) D1 and D2 receptors^47^. Intra-accumbal CARTpt injections also attenuated cocaine self-administration^48^. Non-contingent injection of METH caused significant increases in CARTpt mRNA levels in the NAc of rats euthanized at either 2 hours or one month later^49^. When taken together with our present observations, these results support the notion that CARTpt may play a substantial role in regulating certain aspects of stimulant-induced behaviors^40, 44^ both acutely and after prolonged abstinence.

### Footshock-induced changes in gene expression

In view of the novelty of these observations, it is of interest to briefly discuss the findings that some genes showed significant changes in their expression in animals that had received many non-contingent footshocks (YSR group, see Fig. 6). There is evidence that repeated footshocks can affect DA metabolism in the rodent NAc and dorsal striatum but to different degrees based on the severity of footshocks^50–54^. These studies had investigated perturbances in dopaminergic systems during short periods of time after application of single or multiple footshocks. In the present study, gene expression was assessed after one month of withdrawal and, yet, we still observed significant changes in FMO2, PDK4 and PTPRO mRNA levels in two DA terminal fields. Although the reasons for these changes remain to be determined, these findings suggest that footshock punishment can have substantial transcriptional effects in these brain regions in a fashion that might affect brain metabolism since FMO2 is a member of a class of enzymes (FMO1–5) that are localized in the membranes of endoplasmic reticulum and are involved in metabolizing drugs, regulating fat and cholesterol metabolism, and breaking down of other dietary components^55, 56^. Importantly, the presence of FMOs in the brain^57^ and the increased expression that we observed in the striatum suggests that FMO2 might play a role in the longterm effects of footshocks on the metabolic health of this brain structure. Parenthetically, FMO2 expression is increased by manipulations that increase animal longevity^58^.

Another important metabolic enzyme affected by repeated footshocks in both the striatum and NAc is PDK4, a member of a class of 4 enzymes (PDK1-PDK4)^59, 60^ that are involved in the regulation of the mitochondrial pyruvate dehydrogenase (PDH) complex (PDC)^61, 62^. PDC catalyzes the oxidative decarboxylation of pyruvate to acetyl-coA^63^ and is inactivated via PDK-dependent phosphorylation^59^. PDKs are expressed in the brain^64^ and astrocytes appear to contain higher expression of PDK2 and PDK4 whereas neurons contain higher levels of PDK1^65^. PDKs may be involved in the generation and progression of several neurodegenerative processes that involve inflammation and abnormal mitochondrial functions^66^. Thus, our findings of increased PDK4 mRNA in the NAc and dorsal striatum suggest that footshocks might have activated mechanisms meant to further downregulate PDC function in glial cells to modify biochemical responses to repeated painful stimuli. This notion is consistent with the report that glial responses to inflammatory stimuli were attenuated in PDK4 knockout mice^60^.

Protein phosphorylation/dephosphorylation processes are key regulatory processes that serve to activate or inactivate proteins by the actions of protein kinases and protein phosphatases^67, 68^. Protein tyrosine phosphatases (PTPs) are subdivided into receptor and non-receptor phosphatases and are expressed in the central nervous system^69^. Thus, it was of interest that the mRNA expression of a protein phosphatase, PTPRO, was significantly increased in rats that received large number of non-contingent footshocks. PTPRO was first identified as a membrane protein that is expressed in the brain^70^ and is involved in the regulation of axonal guidance and neurodevelopment^71–73^. The observed increased in PTPRO in the yoked rats suggests that footshocks might have led to activation of mechanisms known to be involved in the regulation of PTPRO-dependent neurodevelopmental processes and, by extension, shock-induced synaptic plasticity in adult rats. In any case, the observations of footshock-induced changes in gene expression are consistent with the idea that adverse consequences can have longterm effects on the brain.

## Conclusion

Our present observations support the notion that accumbal oxytocin is a potentially important neuropeptide in the manifestation of METH addiction. Our data also identify a potential role of striatal CARTpt in regulating rat behaviors after protracted withdrawal from compulsive METH taking. When taken together, the present findings point to a potentially significant role of a diversity of neuropeptides in different stages of METH addiction. This conclusion supports the need for further clinical evaluation of these neuropeptides or their analogs in clinical situations.

## Methods

### Animals and drug treatment

We used male Sprague-Dawley rats (Charles River Labs, Raleigh, NC, USA), weighing 350–400 g in the beginning of the study in all experiments. We housed animals in a humidity and temperature-controlled (22.2 ± 0.2 °C) room with free access to food and water. Our procedures followed the *Guide for the Care and Use of Laboratory Animals* (ISBN 0–309–05377–3) and were approved by the National Institute of Drug Abuse Animal Care and Use Committee.

### Intravenous surgery

We anesthetized rats with ketamine and xylazine (50 and 5 mg/kg, i.p., respectively) and inserted silastic catheters into the jugular veins, as described previously^74^. We attached the catheters to a modified 22-gauge cannula that was mounted to their skulls with dental cement. We injected buprenorphine (0.1 mg/kg, s.c.) one time after surgery to relieve pain and allowed the rats to recover for 5–7 days before methamphetamine self-administration training. During the recovery, training and punishment phases of the experiment, we flushed the catheters every 24–48 h with gentamicin (Butler Schein; 5 mg/ml) and sterile saline.

### Training and punishment phases

We performed the training procedure for methamphetamine self-administration essentially as described^74^. On the first day of training, we brought rats to the self-administration room and chronically housed them in self-administration chambers. Animals had free access to food and water that were available in water bottles and feeders hanging on the walls of all self-administration chambers. We trained rats to self-administer dl-methamphetamine HCl (NIDA) or saline during three 3-h sessions/day (the sessions were separated by 30 min off intervals) for 20 days under a fixed-ratio-1 with 20-s timeout reinforcement schedule. Presses on the retractable active lever activated the infusion pump. Active lever presses were also accompanied by a 5-s compound tone-light cue. Presses on inactive (stationary) lever had no reinforced consequences. We connected the catheters of rats to a modified cannula (Plastics One, Minneapolis, MN) attached to a liquid swivel (Instech Laboratories, Inc., Plymouth Meeting, PA, USA) using a polyethylene-50 tubing that was protected by a metal spring. Rats self-administered methamphetamine for 5 days a week with weekends off. During the 2 days off, rats remained housed in self-administration chambers but were disconnected from intravenous self-administration lines. Also, the levers were retracted and all other cues were removed. Rats self-administered methamphetamine at a dose of 0.1 mg/kg/infusion over 3.5 s (0.1 ml/infusion). To prevent overdose, we limited the number of infusions per 3-h session to 50. Control rats self-administered saline under the same conditions. We started the self-administration sessions at the onset of the dark cycle and sessions began with the insertion of the active lever and the illumination of a red house light that remained on for the duration of the session. At the end of each 3-h session, the house light was turned off, and the active lever was retracted.

During the training plus punishment phase, rats continued methamphetamine self-administration every day (three 3-h sessions/day separated by 30 min off intervals) under the fixed ratio-1 with 20-s timeout reinforcement schedule that was used during training. For methamphetamine-trained rats, 50% of the reinforced lever-presses also resulted in the concurrent delivery of a 0.5-s footshock through the grid floor. We set the initial footshock at 0.18 mA and increased the shock intensity by 0.06 mA to a final value of 0.3 mA (a total of 5 punishment days). Additionally, some control rats were yoked to the shocked animals so that each time animals in the methamphetamine SA group received a contingent shock, these rats also received a non-contingent shock and a saline infusion. Thus, by the end of the behavioral experiments, there were rats yoked to the corresponding shock-sensitive and -resistant rats, namely yoked SS (YSS) and yoked SR (YSR), respectively.

### Withdrawal phase

At the end of the training plus punishment phase, rats were returned to the animal vivarium and individually housed with no access to METH. Intravenous catheters were covered using dust caps and rats had access to home-cage food and water ad libitum. Cue-induced drug craving was then assessed at days 2 and 30 of withdrawal. To test cue-induced craving, rats were brought back to their corresponding SA chambers on the morning of each test. Each test consisted of a single 1-h session during which presses on the drug-associated lever resulted in contingent presentations of the tone and light cues previously paired with METH. However, no METH was available during these tests. Cue-induced drug seeking behavior was assessed using a within-subject design such that all rats tested on day 2 were also tested on day 30 of withdrawal. Animals were euthanized one day after the second cue-induced methamphetamine craving test.

### RNA preparation

Thirty days after cessation of methamphetamine self-administration and footshocks, we euthanized the rats by decapitation with guillotine and isolated NAc and dorsal striatal samples from the brains. We extracted total RNA from individual samples using Qiagen RNeasy Mini kit (Qiagen, Valencia, CA, USA). We assessed RNA integrity using an Agilent 2100 Bioanalyzer (Agilent, Palo Alto, CA, USA). We obtained RNA integrity numbers (RIN, see Table S5) prior to RNA amplification and hybridization to the Affymetrix *GeneChip™ Rat Transcriptome Array 1.0 (RTA 1.0.)* [Thermo Fisher Scientific Inc., Catalog # 902634]. RNA samples showed no degradation with all RINs being greater than 8.4 (see Table S5).

### Transcriptomic profiling using Affymetrix microarrays

The RNA was amplified using Affymetrix labelling based on standard Affymetrix protocols (Affymetrix, Santa Clara, CA, USA). The cRNA and single-stranded cDNA size distribution was determined using an Agilent Bioanalyzer to confirm that the cRNA was the correct size range (200–2000 nt) and the single-stranded cDNA was the correct size (approximately 400 nt). Quality control processes were also used for gene expression analysis including but not limited to hybridization controls, labeling controls, internal control genes (housekeeping controls), global array metrics, and algorithm parameters. Hybridization, labelling, scanning, and data extraction were also performed using standard Affymetrix protocols. Differentially expressed genes were chosen if there were ± 1.7-fold changes (p < 0.05) based on pairwise comparisons using Affymetrix software.

### Quantitative PCR analysis of mRNA levels

Genes were selected for validation by real-time quantitative polymerase chain reaction (qPCR) essentially as previously described [15]. Briefly, individual total RNA from 6 rats per group was reverse-transcribed into cDNA using Advantage RT for PCR kit (Clontech, Mountain View, CA, USA). Gene-specific PCR primers were generated by the LightCycler probe design software v. 2.0 (Roche Biosystems, Indianapolis, IN, USA) and purchased from the Synthesis and Sequencing Facility of Johns Hopkins University (Baltimore, MD USA). High Capacity cDNA Reverse Transcription Kit (Invitrogen, Waltham, MA, USA) was used with a ViiA 7 instrument (Life Technologies, Waltham, MA, USA). The relative amounts of messenger RNA were normalized to means of clathrin, ornithine decarboxylase antienzyme 1 (OAZ1), and tubulin.

### Statistical Analysis

We analyzed the behavioral data with the statistical program SPSS and followed significant effects (p < 0.05) with SPSS post-hoc contrasts with the repeated measures ANOVA. For the training and shock phases, the dependent variables were the number of METH or saline infusions during 20 training days and 5 footshock days. The PCR data were analyzed by one-way ANOVA followed by Fischer’s protected least-significant difference test (PLSD) using StatView (version 4.02, SAS Institute, Cary, NC, USA). The null hypothesis was rejected at p < 0.05.

### Availability of data and materials

Gene expression data have been deposited at the NCBI under the accession #GSE95571.

## Electronic supplementary material

Suplementary Tables
