# Supplementary material for: Compulsive methamphetamine taking in the presence of punishment is associated with increased oxytocin expression in the nucleus accumbens of rats
Source: Sci Rep. 2017 Aug 21;7:8331. doi: 10.1038/s41598-017-08898-8 (PMC5566486; doi:10.1038/s41598-017-08898-8)
Supplement: Supplementary file 1 — Suplementary Tables [file 41598_2017_8898_MOESM1_ESM.pdf]

Compulsive methamphetamine taking in the presence of punishment is associated with increased oxytocin expression in the nucleus accumbens of rats.

Irina N. Krasnova, Maria Carla Gerra, Donna Walther, Subramaniam Jayanthi, Bruce Ladenheim, Michael T. McCoy, Christie Brannock, and Jean Lud Cadet

Supplement Table S1. Classification of genes in the NAc with altered expression in rats receiving footshocks alone.

GenBank Accession	Symbol	Description	Fold Chang YSR vs CT	Fold Change YSS vs CT	Fold Change YSR vs YSS
Metabolism					
NM_001271366	Mki67	marker of proliferation Ki-67	1.05	1.71	-1.63
NM_013015	Ptgds	prostaglandin D2 synthase (brain)	1.34	-1.26	1.68
ENSRNOT00000044617	Ndufb4l1	NADH dehydrogenase (ubiquinone) 1 beta subcomplex, 4-like 1	1.72	1.35	1.27
NM_001106181	Dus2	dihydrouridine synthase 2	-2.01	-2.04	1.01
MicroRNA					
MTA_TR0200016496.mm	Mir3098	microRNA 3098	1.92	3.28	-1.71
NR_037392	Mir344b-1	microRNA 344-b1	-1.04	1.72	-1.79
NR_037373	Mir3580	microRNA mir-3580	-1.74	-1.53	-1.13
NR_032278_2	Mir434	rno-mir-3543	1.46	-1.51	2.21
Oxygen Transport					
NM_033234	Hbb	hemoglobin	-1.28	-2.11	1.65
NM_013096	Hba1	hemoglobin, alpha 1	-1.17	-1.77	1.52
ENSRNOT00000048977	Hba2	hemoglobin, alpha 2	-1.21	-1.67	1.38
NM_198776	Hbb-b1	hemoglobin, beta adult major chain	-1.61	-3.63	2.26
Protein Kinase/Phosphatase					
NM_017336	Ptpro	protein tyrosine phosphatase, receptor type, O	1.66	-1.09	1.82
NM_031769	Pdxk	pyridoxal (pyridoxine, vitamin B6) kinase; similar to pyridoxal (pyridoxine, vitamin B6) kinase; pyridoxal kinase-like	1.74	1.64	1.06
NM_053551	Pdk4	pyruvate dehydrogenase kinase, isozyme 4	1.84	1.15	1.59
Proteolysis					
NM_020091	Cym	chymosin	-2.61	1.65	-4.30
Signal Transduction					
NM_001000365	Olr748	olfactory receptor 748	-1.37	-1.72	1.26
NM_001106431	Stoml3	stomatin (Epb7.2)-like 3	-1.31	1.40	-1.83
Transcription					
NM_019328	Nr4a2	nuclear receptor subfamily 4, group A, member 2	1.72	1.79	-1.04

Supplement Table S2. Genes with altered expression the NAc of rats receiving comparable number of footshocks in the presence or absence of METH SA.

GenBank Accession	Symbol	Description	Fold Change SR vs YSR	Fold Change SS vs YSS
Cell Adhesion				
NM_001034009	Mcam	melanoma cell adhesion molecule	1.55	1.72
NM_022285	Hapln2	hyaluronan and proteoglycan link protein 2	1.34	1.73
Cell Growth				
NM_030868	Nov	nephroblastoma overexpressed	-1.56	-2.25
Hormonal				
NM_012829	Cck	cholecystokinin	-2.28	-2.49
NM_012996	Oxt	oxytocin/neurophysin 1 prepropeptide	5.98	-1.00
NM_016992	Avp	arginine vasopressin	1.80	-1.08
Immunity				
NM_012752	Cd24	CD24 molecule	-1.14	-1.66
NM_001191752	Ccdc77	coiled-coil domain containing 77	3.00	2.42
NM_016994	C3	complement component 3	1.07	1.68
Metabolism				
NM_022224	Pter	phosphotriesterase related	1.04	-1.68
NM_133395	Serinc5	serine incorporator 5	1.65	1.66
MicroRNA				
NR_037370	Mir1912	microRNA 1912	-1.79	-1.23
MTA_TR1900001750.mm	Mir3970	microRNA 3970	1.09	-1.75
NR_032296	Mir770	microRNA 770	1.69	2.36
NR_031933	Mir219-2	microRNA mir-219-2	1.30	1.84
NR_032119	Mir543	microRNA mir-543	1.09	1.69
NR_032286	Mir598	microRNA mir-598	-1.18	1.81
MTA_TR0X00002108.mm	Mir7092	microRNA mir-7092	-2.41	-1.22
NR_032286_2	Mir598	microRNA mir-598	-1.18	1.81
Protein Kinase/Phosphatase				
NM_017336	Ptpro	protein tyrosine phosphatase, receptor type, O	-1.73	-1.10
NM_053551	Pdk4	pyruvate dehydrogenase kinase, isozyme 4	-1.68	1.12
NM_001193568	Sgk1	serum/glucocorticoid regulated kinase 1	-1.75	1.19
Proteolysis				
NM_020091	Cym	chymosin	1.90	-3.94
Signal Transduction				
NM_012798	Mal	mal, T-cell differentiation protein	1.71	1.41
NM_022589	Tspan2	tetraspanin 2	1.50	1.65
NM_001271297	Spock1	sparc/osteonectin, cwcv and kazal-like domains proteoglycan (testican) 1	1.73	1.47
NM_001106227	Tmem160	transmembrane protein 160	1.65	1.15
NM_001109426	Tmem88b	transmembrane protein 88B	1.71	1.79
NM_181381	Abcg2	ATP-binding cassette, subfamily G, member 2	2.27	1.84
Synaptic Plasticity				
NM_001107251	Fezf2	Fez family zinc finger 2	-1.24	-1.82
NM_001109306	Satb2	SATB homeobox 2	-1.15	-1.86
Transcription				
NM_001106571	Lhx2	LIM homeobox 2	-1.17	-1.73

NM_019328	Nr4a2	nuclear receptor subfamily 4, group A, member 2	-1.95	-1.97

Supplement Table S3. Classification of genes with altered expression in the striatum of rats receiving non-contingent footshocks.

GenBank Accession	Gene Symbol	Description	Fold Change YSR vs CT	Fold Change YSS vs CT	Fold Change YSR vs YSS
Biological Function					
NM_001106087	Tex15	testis expressed 15	1.21	-1.49	1.80
NM_001191922	Shisa6	shisa family member 6	-1.48	1.97	-2.92
Calcium Ion Binding					
NM_001108586	Rcn1	reticulocalbin 1, EF-hand calcium binding domain	-1.29	-1.96	1.52
NM_001276720	Otof	otoferlin; Unannotated AceView Transcript	-1.23	1.52	-1.86
NM_022302	Necab1	N-terminal EF-hand calcium binding protein 1	1.18	1.70	-1.44
Cell Adhesion					
NM_001169129	Pcdh19	protocadherin 19	-1.16	1.82	-2.11
NM_053393	Cdh8	cadherin 8	-1.36	-1.73	1.27
XM_001056235.6	Pcdhb22	protocadherin beta 22	-1.49	-1.74	1.17
Cell Differentiation					
XM_008768150.2	Slfn5	schlafen family member 5	1.57	-1.14	1.78
DNA Binding					
XM_008761346.2	Hist2h3c2	histone cluster 2, H3c2	1.09	-1.87	2.04
Membrane Structure					
NM_001044269	Tmem19	transmembrane protein 196	1.17	2.29	-1.96
XM_006230914.2	LOC102548568	vegetative cell wall protein gp1-like	1.90	-1.14	2.18
Metabolism					
NM_001013084	Akr1b1	aldo-keto reductase family 1	-2.91	-1.29	-2.25
NM_001080756	Car12	carbonic anyhydrase 12	2.32	1.21	1.92
NM_001105987	Tatdn3	TatD DNase domain containing 3	-2.27	-1.02	-2.24
NM_001106181	Dus2	dihydrouridine synthase 2	-1.79	-1.81	1.01
NM_001108820	Gla	galactosidase, alpha	1.68	1.25	1.34
NM_001142915	Plod2	procollagen lysine, 2-oxoglutarate 5-dioxygenase 2	1.73	-1.17	2.01
NM_001145163	Ube2ql	ubiquitin-conjugating enzyme E2Q family-like 1	-1.52	1.74	-2.64
NM_020091	Cym	chymosin	-1.75	1.55	-2.72
NM_023025	Cyp2j4	cytochrome P450, family 2, subfamily j	-1.10	-1.78	1.62
NM_031655	Lxn	latexin	1.17	2.04	-1.74
NM_031776	Gda	guanine deaminase	-1.07	2.27	-2.42
NM_053562	Rpe65	retinal pigment epithelium 65	1.89	-1.06	2.02
NM_053850	Blvra	biliverdin reductase A	-1.06	1.59	-1.68
NM_144737	Fmo2	flavin containing monooxygenase 2	2.69	1.11	2.42
XM_008762861.2	LOC100910708	aldose reductase-related protein 1-like	-2.81	-1.16	-2.41
MicroRNA					
NR_032119	Mir543	microRNA mir-543	1.24	1.84	-1.49

NR_105854	Mirlet7k	microRNA let-7k	-1.34	1.25	-1.67
NR_130330	Mir6691	microRNA 669l	-2.11	-1.14	-1.85
Oxygen Transporter Activity					
NM_033234	Hbb	hemoglobin, beta	-1.12	-2.13	1.90
NM_198776	Hbb-b1	hemoglobin, beta adult major chain	1.02	-3.33	3.39
Protein Binding					
NM_001025682	Cdr2	cerebellar degeneration-related protein 2	1.62	-1.70	2.76
NM_001048215	Kirrel3	kin of IRRE like 3 (Drosophila)	-1.26	-1.74	1.39
NM_001100172	Kctd8	potassium channel tetramerization domain containing 8	1.03	-1.9	1.95
NM_001108454	Cpne7	copine VII	-1.24	2.35	-2.91
NM_001109403	C1ql3	complement component 1, q subcomponent-like 3	-1.62	2.43	-3.92
NM_001126093	Pcp4l1	Purkinje cell protein 4-like 1	1.09	-2.31	2.51
NM_001191966	Napb	N-ethylmaleimide-sensitive factor attachment protein,	-1.16	1.81	-2.09
NM_019354	Ucp2	uncoupling protein 2 (mitochondrial, proton carrier)	1.49	-1.48	2.21
NM_031688	Sncg	synuclein, gamma	1.71	-1.28	2.18
XM_017593021.1	Frmd4b	FERM domain containing 4B	-1.17	-2.12	1.81
Protein Phosphorylation & Dephosphorylation					
XM_017593903.1	Sgk3	serum/glucocorticoid regulated kinase family, member 3	1.34	-1.37	1.83
NM_001005871	Atp2b4	ATPase, Ca++ transporting, plasma membrane 4	-1.81	2.43	-4.41
NM_001108951	AK5	similar to adenylate kinase 5 isoform 1	1.06	2.00	-1.89
NM_017336	Ptpro	protein tyrosine phosphatase, receptor type, O	2.19	-1.03	2.25
NM_031571	Acvr2a	activin A receptor, type IIA	1.07	2.23	-2.08
NM_053551	Pdk4	pyruvate dehydrogenase kinase, isozyme 4	2.57	1.49	1.73
NM_182842	Camk1g	calcium/calmodulin-dependent protein kinase IG	-1.30	2.20	-2.86
Signal Transduction					
XM_002729002.3	Iqgap2	IQ motif containing GTPase activating protein 2	1.38	-1.41	1.94
XM_003748966.4	LOC100912217	olfactory receptor 10A3-like	-1.30	1.28	-1.66
NM_001002830	Rasl11b	RAS-like family 11 member B	1.08	1.89	-1.75
NM_001004263	Itgb6	integrin, beta 6	1.27	-1.71	2.18
NM_001014092	Paqr5	progestin and adipoQ receptor family member V	1.60	-1.03	1.65
NM_001014232	Cnrip1	cannabinoid receptor interacting protein 1	-1.16	1.87	-2.18
NM_001047084	Rgs18	regulator of G-protein signaling 18	-1.29	1.94	-2.50
NM_001106124	Slc39a	solute carrier family 39 (zinc transporter)	1.52	2.07	-1.36
NM_001109295	Cplx3	complexin 3	-1.38	1.98	-2.74
NM_001130575	Rspo2	R-spondin 2	-1.11	1.84	-2.05
NM_001166578	Cnih3	cornichon family AMPA receptor auxiliary protein 3	-1.33	1.68	-2.23
NM_001191953	Pgm5	phosphoglucomutase 5	-1.32	-1.76	1.33
NM_001257349	Gng2	guanine nucleotide binding protein (G protein)	-1.40	2.11	-2.95
NM_001287022	Rgs21	regulator of G-protein signaling 21	1.08	1.83	-1.69
NM_012827	Bmp4	bone morphogenetic protein 4	1.61	-1.67	2.69

NM_012832	Chrna7	cholinergic receptor, nicotinic, alpha 7 (neuronal)	-1.03	1.67	-1.72
NM_012853	Htr4	5-hydroxytryptamine (serotonin) receptor 4	-1.16	1.54	-1.79
NM_013069	Cd74	Cd74 molecule, major histocompatibility complex	1.59	-1.53	2.43
NM_013099	Mc4r	melanocortin 4 receptor	1.05	3.11	-2.95
NM_017295	Gabra5	gamma-aminobutyric acid (GABA) A receptor, alpha 5	-1.82	1.77	-3.21
NM_017296	Kcnj2	potassium inwardly-rectifying channel, subfamily J	-1.66	-1.38	-1.21
NM_017310	Sema3a	sema domain, immunoglobulin domain (Ig), short basic do	-1.04	2.68	-2.8
NM_017362	Chrm5	cholinergic receptor, muscarinic 5	-1.13	3.05	-3.45
NM_019348	Sstr2	somatostatin receptor 2	-1.02	1.79	-1.83
NM_019374	Pdyn	prodynorphin	-1.23	1.09	-1.35
NM_022202	Grm8	glutamate receptor, metabotropic 8	-1.11	2.12	-2.36
NM_022266	Ctgf	connective tissue growth factor	-1.24	2.08	-2.58
NM_022632	Slit2	slit homolog 2 (Drosophila)	2.03	2.21	-1.09
NM_031602	Kcnj10	potassium inwardly-rectifying channel, subfamily J	1.17	-1.77	2.08
NM_031823	Wfs1	Wolfram syndrome 1 (wolframin)	-1.22	2.86	-3.49
NM_053324	Syt9	synaptotagmin IX	-1.29	-2.25	1.74
NM_053613	Rtn4r	reticulon 4 receptor	-1.19	1.88	-2.24
NM_053721	Rasgrf2	RAS protein-specific guanine nucleotide-releasing factor	-1.06	3.09	-3.27
NM_080693	Cacng5	calcium channel, voltage-dependent, gamma subunit 5	-1.12	2.45	-2.74
NM_198791	Tlr3	toll-like receptor 3	1.82	-1.41	2.56
NM_201417	Rxfp1	relaxin/insulin-like family peptide receptor 1	-1.00	2.41	-2.41
NM_207616	Hyal1	hyaluronoglucosaminidase 1	1.94	1.05	1.84
Transcription					
XM_017591165.1	Mef2c	myocyte enhancer factor 2C; similar to myocyte enhancer	1.21	1.80	-1.49
NM_001034002	Yap1	yes-associated protein 1	1.37	-1.23	1.69
NM_001106895	Tfap2d	transcription factor AP-2 delta	-1.06	1.65	-1.76
NM_001134706	Hmgn5b	high mobility group nucleosome binding domain 5B	1.43	-1.18	1.68
NM_001191846	Foxo1	forkhead box O1;	-1.29	-2.74	2.12
NM_013058	Id3	inhibitor of DNA binding 3	1.14	-1.77	2.02
NM_017149	Meox2	mesenchyme homeobox 2	-1.35	-1.85	1.37
NM_019328	Nr4a2	nuclear receptor subfamily 4, group A, member 2	1.03	6.74	-6.56
NM_024360	Hes1	hes family bHLH transcription factor 1	1.67	1.15	1.45
NM_031059	Msx1	msh homeobox 1	2.38	-1.02	2.42
NM_138838	Pou3f1	POU class 3 homeobox 1	-1.60	-1.81	1.13
Unknown					
XM_008770180.1	Nwd2	NACHT and WD repeat domain containing 2	1.01	2.04	-2.02
NM_001009701	Itfg3	integrin alpha FG-GAP repeat containing 3	1.10	-1.69	1.85
NM_001024369	Ypel4	yippee-like 4 (Drosophila)	-1.16	1.72	-2.00

Supplement Table S4. Classification of striatal genes with altered expression after footshocks in the presence or absence of METH SA.

GenBank Accession	Gene Symbol	Description	Fold Change SR vs YSR	Fold Change SS vs YSS
Biological Function				
NM_001034011	Sft2d2	SFT2 domain containing 2	-1.73	-1.19
NM_001106087	Tex15	testis expressed 15	-1.25	2.26
NM_001107445	Hspa12a	heat shock protein 12A	1.75	1.05
NM_001191752	Ccdc77	coiled-coil domain containing 77	2.14	2.75
Calcium Ion Binding				
NM_001106879	Efhb	EF hand domain family, member B	-2.64	1.58
Cell Differentiation				
NM_001014163	Cend1	cell cycle exit and neuronal differentiation 1	2.10	1.15
DNA Binding				
NM_001013181	Zbtb16	zinc finger and BTB domain containing 16	-2.04	-1.41
Immunity				
XM_008760831.1	C7	complement C7	-5.16	-1.04
Metabolism				
NM_001013084	Akr1b1	aldo-keto reductase family 1	2.85	1.22
NM_001048042	Nmnat2	nicotinamide nucleotide adenylyltransferase 2	1.66	-1.04
NM_001080756	Car12	carbonic anyhydrase 12	-2.12	1.30
NM_001135756	Galntl6	polypeptide N-acetylgalactosaminyltransferase-like 6	1.79	1.05
NM_001142915	Plod2	procollagen lysine, 2-oxoglutarate 5-dioxygenase 2	-1.65	1.33
NM_001191674	Pld5	phospholipase D family, member 5	1.65	-1.02
NM_001258237	Ace3	angiotensin I converting enzyme (peptidyl-dipeptidase A	-2.55	2.22
NM_012886	Timp3	TIMP metallopeptidase inhibitor 3	-1.97	1.64
NM_013096	Hba1	hemoglobin, alpha 1	-1.04	1.71
NM_016994	C3	complement component 3	-1.11	1.93
NM_020091	Cym	chymosin	1.86	-2.53
NM_021776	Ecel	endothelin converting enzyme-like 1	1.77	1.05
NM_022215	Gpd1	glycerol-3-phosphate dehydrogenase 1 (soluble)	-2.29	-1.78
NM_130744	Cygb	cytoglobin	2.35	-1.02
NM_144737	Fmo2	flavin containing monooxygenase 2	-2.54	1.30
XM_006247643.3	LOC102556346	angiotensin-converting enzyme-like	-3.72	4.15
XM_008762861.2	LOC100910708	aldose reductase-related protein 1-like	2.79	1.10
MicroRNA				
NR_035479	Mir1954	microRNA 1954	1.84	-1.90
NR_035497	Mir1970	microRNA 1970	-1.67	-1.56
NR_037339	Mir466d	microRNA mir-466d	1.17	-2.19
NR_105756	Mir6338	microRNA mir-6338	1.92	1.08
NR_106128	Mir7668	microRNA mir-7668	-1.10	-1.70
NR_130330	Mir6691	microRNA 669l	1.75	-1.16
Oxygen Transporter Activity				
NM_033234	Hbb	hemoglobin, beta	-1.35	1.55
NM_198776	Hbb-b1	hemoglobin, beta adult major chain	-1.77	2.46
Protein Binding				

NM_001025682	Cdr2	cerebellar degeneration-related protein 2	-1.97	1.94
NM_001108750	Cpne8	copine VIII	1.94	1.09
NM_001109210	Cbln4	cerebellin 4 precursor	1.47	-1.68
NM_013060	Id2	inhibitor of DNA binding 2	-1.75	-1.17
NM_017110	Cartpt	CART prepropeptide	1.87	-1.37
NM_031688	Sncg	synuclein, gamma	-2.37	1.51
XM_017593021.1	Frmd4b	FERM domain containing 4B	-1.05	1.87
XM_575125.6	Wdr38	WD repeat domain 38	-1.70	1.10
Protein Phosphorylation & Dephosphorylation				
NM_001108055	Ak7	adenylate kinase 7	-1.81	-1.05
NM_001193568	Sgk1	serum/glucocorticoid regulated kinase 1	-2.52	-1.21
NM_031004	Acta2	actin, alpha 2, smooth muscle, aorta	1.82	-1.02
NM_053551	Pdk4	pyruvate dehydrogenase kinase, isozyme 4	-2.89	-1.41
Signal Transduction				
XM_575397.7	Wnt2	wingless-type MMTV integration site family member 2	1.01	-1.78
NM_001000068.1	Olr1024	olfactory receptor 1024	1.06	-1.79
NM_001001019	Olr349	olfactory receptor 349	1.78	1.07
NM_001004263	Itgb6	integrin, beta 6	-1.89	1.83
NM_001008555	Slc10a	solute carrier family 10, member 4	1.98	1.35
NM_001012116	Spag1	sperm associated antigen 1	-1.76	1.21
NM_001014071	Errfi1	ERBB receptor feedback inhibitor 1	-1.70	-1.00
NM_001024241	Gpr139	G protein-coupled receptor 139	3.27	1.29
NM_001024979	Gpatch4	G patch domain containing 4	-2.02	-1.52
NM_001169138	Thbs2	thrombospondin 2	-2.52	1.41
NM_012665	Syt2	synaptotagmin II	1.69	1.28
NM_012827	Bmp4	bone morphogenetic protein 4	-1.42	2.18
NM_013036	Sstr4	somatostatin receptor 4	1.07	-1.66
NM_019374	Pdyn	prodynorphin	1.79	1.03
NM_023970	Trpv4	transient receptor potential cation channel	-1.85	1.14
NM_053296	Glrb	glycine receptor, beta	1.77	-1.08
NM_053521	Slc5a7	solute carrier family 5 (sodium/choline cotransporter)	1.67	1.41
NM_080693	Cacng5	calcium channel, voltage-dependent, gamma subunit 5	2.31	-1.69
NM_130816	Fgf11	fibroblast growth factor 11	2.01	1.05
NM_181381	Abcg2	ATP-binding cassette, subfamily G (WHITE), member 2	1.97	1.82
NM_198791	Tlr3	toll-like receptor 3	-1.72	1.86
Transcription				
XM_017596208.1	Rbms3	RNA binding motif, single stranded interacting protein	1.71	1.17
XM_006247958.3	Znf750	zinc finger protein 750	-1.87	1.23
NM_001005882	Rbm47	RNA binding motif protein 47	-1.93	1.25
NM_012943	Dlx5	distal-less homeobox 5	1.82	1.03
NM_031059	Msx1	msh homeobox 1	-2.25	1.22
Unknown				
XM_017599433.1	Fam47e	family with sequence similarity 47, member E	-2.02	1.24
XM_017596811.1	KIAA2012	similar to KIAA2012 protein	-1.71	1.21
NM_001106527	Tspyl3	TSPY-like 3	1.65	-1.13

Supplement Table S5. RNA Integrity Number (RIN) of samples used in Affymetrix array

	RIN Number	
Sample	Nucleus accumbens	Striatum
CT 1	8.5	9.3
CT 2	8.7	9.1
CT 3	8.8	9.3
CT 4	8.7	9.2
CT 5	8.7	8.8
CT 6	8.6	8.6
SR 1	8.6	9.2
SR 2	8.6	9.1
SR 3	8.6	8.9
SR 4	8.9	9.0
SR 5	8.7	9.3
SR 6	8.6	9.3
SS 1	8.4	8.9
SS 2	8.8	8.9
SS 3	8.9	8.8
SS 4	8.8	8.7
SS 5	8.7	8.8
SS 6	8.6	8.9
YSR 1	8.4	9.3
YSR 2	8.5	8.8
YSR 3	8.7	9.2
YSS 1	8.6	9.2
YSS 2	8.7	9.2
YSS 3	8.5	9.0

RIN takes into account the values for 28S, 18S and small ribosomal RNAs compared to one another as well as the total signal. RIN is used for assigning integrity values to RNA measurements. The higher RIN correlates with better outcomes of RT-PCR and microarrays. Schroeder et. al. (2006) demonstrated that the RIN greater than 6 to be of high quality. Note, the RIN values are all equal or greater than 8.4
.

Schroeder, A. et al. (2006) The RIN: an RNA integrity number for assigning integrity values to RNA measurements. BMC Mol. Biol. 7: 3.
